# Universal credit receipt among users of secondary mental health services: Findings from a novel data linkage

**DOI:** 10.23889/ijpds.v8i2.2224

**Published:** 2023-09-14

**Authors:** Sharon Stevelink, Matthew Hotopf, Ira Madan, Ava Phillips, Ray Leal, Nicola T Fear

**Affiliations:** 1 King's College London, London, United Kingdom; 2 Guy's St Thomas NHS Foundation Trust, London, United Kingdom

## Objectives

People with mental disorders are likely to be overrepresented among Universal Credit (UC) recipients. Despite this, individual level data is lacking on mental disorder diagnosis by UC status, and the intersectionality of different socio-demographic characteristics. Associations were explored between UC receipt, socio-demographic and diagnostic characteristics among mental health service users.

## Method

A novel data source consisting of linked electronic mental healthcare records and Department for Work and Pensions administrative benefits records of patients accessing a large mental healthcare service provider in South London was used. Benefits records were restricted to those that covered years 2013-2019. Only working-age patients were included (n=120,000).

## Results

Preliminary results indicated that of the 120,000 working-age patients with linked data, 38,000 had received UC at some point between 2013-2019. Most UC recipients were allocated to a conditionality regime that required them to look for work, followed by those who did not have to meet any work-related requirements. Adjusted analyses indicated that UC recipients were more likely to be male, younger, lived in more deprived areas, and were from a non-White ethnic background. Interestingly, having a recorded psychiatric diagnosis meant that patients were less likely to have received UC.

## Conclusion

Possible explanations for the findings will be discussed. In the longer term, our findings have the potential to impact welfare and public health policies, as well as patient care.

